# A Method for Determining the Content of Glycoproteins in Biological Samples

**DOI:** 10.3390/molecules21121625

**Published:** 2016-11-26

**Authors:** Yang Gao, Duoduo Xu, Hongyue Li, Xianling Yang, Mingxing Wang, Qipin Gao

**Affiliations:** 1 Jilin Key Laboratory of Macromolecules in Chinese Drugs, Changchun University of Chinese Traditional Medicine, Changchun 130117, Jilin, China; gaoyang-1979@hotmail.com (Y.G.); czxuduoduo@163.com (D.X.); 18311164062@163.com (H.L.); cc_wmx@163.com (M.W.); 2 Jilin Institute For Drug Control, Changchun 130033, Jilin, China; vivienyang22@sina.com

**Keywords:** glycoprotein, iodine substitution, ICP-MS, biological sample, quantification

## Abstract

The glycoprotein purified from the mycelium extract of *Tremella fuciformis* was marked with iodine through the iodine substitution reaction. The content of iodine, which is indicative of the amount of the marked tremella glycoprotein (ITG), was detected with Inductively coupled plasma mass spectrometry (ICP-MS). The method was found to be stable, sensitive, and accurate at detecting the content of iodine-substituted glycoprotein, and was used in the quantitative analysis of biological samples, including blood and organs. Different biological samples were collected from rats after oral administration of ITG, and were tested for iodine content by ICP-MS to calculate the amount of ITG in the samples. The results suggested that ICP-MS is a sensitive, stable, and accurate method for detection of iodinated glycoproteins in blood and organs.

## 1. Introduction

Glycoproteins are important biological macromolecules, with a wide variety of biological functions. Glycoproteins possess almost no alienation of metabolism and biological toxicity, and rarely interact with other drugs. Because of the above characteristics, glycoproteins have become highly valued in life sciences research and drug development. However, since the structure of glycoproteins is very complex, they are difficult to separate and purify. The lack of a simple, sensitive method for detecting glycoproteins in biological samples makes bioavailability and pharmacokinetic studies of glycoproteins very demanding, seriously restricting research.

Pharmacokinetics is related to drug absorption, distribution, metabolism, and elimination (ADME). It is used in the studies of drug mechanisms of action, for designing and optimizing dosage regimens, and for quality control. Studies of bioavailability and pharmacokinetics of glycoproteins are relatively difficult, as ideal quantification methods for biological samples are lacking. Methods used for detection of glycoproteins in biological samples include chromatography [1], radioactive isotope labeling [2,3], fluorescence assay [1,4], and the ELISA method [5]. Because of the complex structures and large molecular weights of glycoproteins, and low purity of samples, the sensitivity of chromatographic, fluorescence labeling methods is usually insufficient for pharmacokinetic studies, especially given the low content of glycoproteins in biological samples and strong interference of other substances present. Bioassays require the preparation of specific antibodies targeting the glycoproteins studied, which is a long and not always successful procedure. Isotope labeling methods are more sensitive and more widely applicable. However, experimental conditions of isotopic labeling are demanding and costly, and the procedure carries the risk of radioactive pollution. Defining and establishing a simple, convenient, tracer-based determination method, is one of the key scientific problems in bioavailability, pharmacokinetics, and mechanistic studies of glycoproteins. 

Glycoproteins can be iodinated if they contain tyrosine, and this property was used to label proteins with isotopic iodine. Stable but less biologically active iodinated fluorescein derivatives of polysaccharides have also been prepared [3]. Labeling complex carbohydrate molecules, including polysaccharides, glycoproteins, and glycolipids with radioactive isotopes of iodine is a classic and widely used method in pharmacokinetic studies of these compounds [6,7,8,9].

Inductively coupled plasma mass spectrometry (ICP-MS) is an analytical technique developed in recent years. ICP-MS has the potential to replace the traditional inorganic analysis techniques such as inductively coupled plasma spectroscopy and graphite furnace atomic absorption in qualitative and quantitative analysis of inorganic elements. In addition, ICP-MS can detect halogen elements with high sensitivity and robustness. Therefore, iodination of sugar complexes, combined with ICP-MS, is expected to establish a new, simple, reliable, and widely applicable method for identification and quantification of labeled sugar complexes in biological samples. This paper reports the preparation of iodine substituted glycoproteins and detection of iodine content using ICP-MS in rat blood and organs after oral administration of glycoproteins. 

## 2. Methods

### 2.1. Reagents and Materials

The mycelium extract of *Tremella fuciformis* was purchased from Jilin Xinhua Medicine Ltd. (Tonghua City, Jilin, China). Standards for d-mannose (≥99%), d-glucuronic acid (≥99%), and bovine serum albumin (≥99%) were obtained from Sigma (St. Louis, MO, USA). Sephadex G-50, Sephadex G-100, and standards of dextrans were obtained from Pharmacia (Stockholm, Sweden). Bio-Rad protein assay reagents were purchased from Bio-Rad (Berkeley, CA, USA); all other chemicals were of reagent grade and used as such. Cell culture (RPM 450) was purchased from Thermo, Rockford, IL, USA. The no iodine feeds of SPF rat were purchased from Beijing Aokexieli Feed Co., Ltd. (Beijing, China).

### 2.2. Analysis of Physicochemical Properties

The total carbohydrates, uronic acids, and proteins were quantified using phenol-sulfuric acid [10], m-hydroxydiphenyl [11] and Bio-Rad protein methods, respectively. Glucose (Glc), glucuronic acid (GlcA), and bovine serum albumin (BSA) were used as standards, respectively. The molecular weight of the sample was analyzed by high-performance liquid chromatography (Shimadzu LC-2010, Kyoto, Japan) using an OH-park column equilibrated with 0.7% sodium sulfate, and the calibration curves were obtained using dextran as a standard. The molecular weight was calculated by GPC software (the National Institute for the Control of Pharmaceutical and Biological Products of China, Beijing). The sugar components were analyzed by converting the sugars into 1-phenyl-3-methyl-5-pyrazolone (PMP) derivatives [12] which were detected by HPLC. HPLC was carried out at a Shimadzu 2010 instrument equipped (Tokyo, Japan) with a C18 column. The amino acids were analyzed using an S-433D (Sykam, Eresing, Germany) automatic amino acid analyzer. The FT-IR spectra were acquired using Bruker Vertex 70 FTIR (Bruker, Germany). The samples were pressed into KBr pellets and the spectra were recorded in transmittance mode over the frequency range of 4000–400 cm^−1^.

### 2.3. Preparing the Glycoprotein from the Extract of T. fuciformis

**Figure 1:**
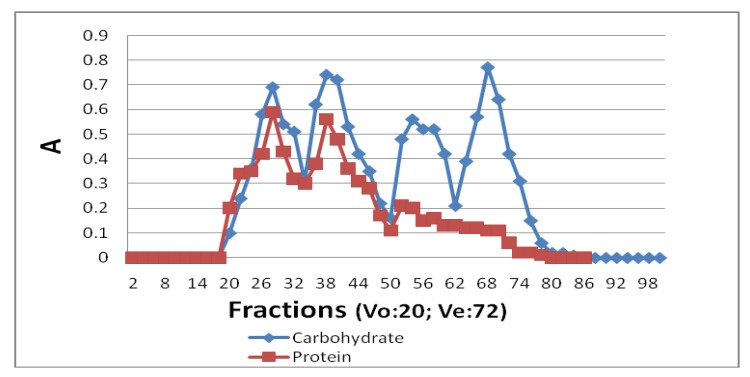
Gel-chromatography of crude polysaccharides (P) on Sephadex G-100.

The mycelium extract of *T. fuciformis* was dissolved in water, and the solution was centrifuged to remove the solid residue. The aqueous solution was concentrated before being precipitated with 70% ethanol, which was removed by centrifuge (4500 rpm, 15 min). The precipitated material was lyophilized to afford the crude polysaccharides (P). Afterwards the polysaccharides (15 g) were applied to a Sephadex G-100 column (Pharmacia, Stockholm, Sweden; 80 cm × 10 cm) and washed with water. The eluted fractions (15 mL) were collected and carbohydrates, uronic acids, and proteins were detected in each fraction. According to the elution pattern (Figure 1), the fractions 18–34, 35–50, 51–62, and 63–80 were collected and lyophilized to get the combined A, B, C, and D fractions. Properties of the fractions are listed in Table 1.

Fraction B was used in the study as tremella glycoprotein (TGP, 4.8 g) according to the analysis.

### 2.4. Iodine Substitution Reaction

The iodine substitution was performed according the report of Klaus Keck [9] with slight modifications. Tremella glycoprotein (TGP, 200 mg) was dissolved in 5 mL of water, and 1 mL (0.5 mol/L) NaI and 1 mL (0.1 g/mL) chloramine T solution were added to start the reaction. The reaction mixture was shaken for 1 min, and 1 mL (0.1 g/mL) sodium metabisulfite and 2 mL (0.1 g/mL) potassium iodide were added, while mixing, to stop the reaction. Reaction mixture was centrifuged (5000 r/min, 10 min), and the supernatant was collected and freeze dried to get iodine-substituted tremella glycoprotein (CITG).

### 2.5. Purification of CITG by Gel Chromatography

**Figure 2:**
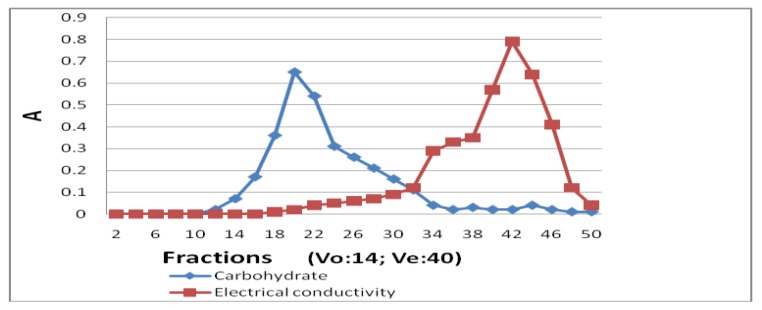
Purification of ITG on Sephadex G-50 column.

CITG (200 mg) was dissolved in 10 mL pure water and the sample solution was applied to a Sephadex G-50 column (40 cm × 5 cm), and eluted with water. According to the elution pattern (Figure 2) as described by the carbohydrate content and electrical conductivity in each collected fraction (12 mL/tube) and the volume of water inside and outside of the column, fractions 18–32 were collected, concentrated, and freeze dried to remove the remaining reagents and obtain the purified iodine-substituted tremella glycoprotein (ITG). 

### 2.6. Stability Study of ITG in Cell Culture

ITG (100 mg) was dissolved in 50 mL of cell culture (RPMI 1640) and put in a shaking table at 37 °C. Every hour, 10 mL of the cell culture was taken out to pass through a Sephadex G-50 column (30 cm × 1.2 cm) and the first 5 mL and the second 5 mL of the eluent, which are close to excluded volume, were collected to analyze the content of iodine by ICP-MS.

### 2.7. Digestion of Samples

ITG or biological samples were weighted, transferred to a headspace sampling bottle (10 mL), and 4 mL ultrapure water, 3 mL 1% tetramethylammonium hydroxide (TMAH), and 0.1 mL H_2_O_2_ were added, in that order. Headspace sample bottles were closed, put in an ultrasonic water bath under 80 °C, and sonicated for 30 min to digest the sample. After digestion, samples were filtrated with 0.45 µm filter membrane and diluted for ICP-MS determination.

### 2.8. Detection of the Iodinated Glycoprotein with ICP-MS

ICP-MS was performed using Agilent 7700 ICP-MS. Mass Spectrometry (Santa Clara, CA, USA) tuned liquid: 7Li, 59Co, 89Y, 140Ce, 205Ti was used. The internal standard was 1 mg/mL indium solution. The conditions of detection are presented in Table 2. Digested sample solution was diluted before injection. The ICP-MS system was washed for 90 s between injections to reduce the memory effect. 

### 2.9. Preparation of Blood and Organ Samples

The rats (190–210 g) were obtained from Jilin University, College of Pharmacy, Changchun, China, certificate of conformity for the SCXK, (2012-0003). On the fifth day of normal feeding, the rats were deprived of food, but allowed free access to bottled pure water for 12 h and randomly divided into control and oral dosage groups. Each group consisted of three rats. The oral dosage group was administered ITG (200 mg/rat dissolved in 2 mL pure water), whereas the control group received the same volume of pure water. The blood samples (0.5 mL) were collected from the eye vein of anesthetized rats with diethyl ether at 1, and 2 h after administration of ITG. Afterwards, at 3 h after administration, the rats were anesthetized with diethyl ether and then sacrificed to obtain the blood and organ samples which were freeze-dried immediately and powdered and 100 mg powdered samples were used for further analysis. All data are expressed as mean ± SE.

## 3. Results

### 3.1. Preparation of Glycoprotein Samples

The glycoprotein samples used in the experiment were prepared in our laboratory since no suitable samples were commercially available. The TGP showed a single peak with the molecular weight of 10.4 KDa in the GPC analysis and contained about 11% of protein, which could not be removed from TGP using the Savage method or gel-filtration chromatography (data not shown). Our analysis indicated that TGP is a water-soluble glycoprotein (or glycopeptide), whose sugar component contained mannose, glucose, galactose, arabinose, and glucuronic acid in the molar ratio of 1.0:0.3:0.3:0.2:0.2. The protein portion of TGP was composed of 14 amino acids (weight percent): arginine (18.1%), lysine (10.9%), phenylalanine (10.3%), tyrosine (9.8%), histidine (8.2%), glycine (7.2%), glutamate (6.6%), tryptophan (6.6%), alanine (5.9%), hydroxyproline (5.0%), proline (4.9%), aspartic acid (4.2%), methionine (1.2%), and isoleucine (1.1%).

### 3.2. Iodination of Glycoprotein

Iodination of TGP was catalyzed by chloramine T, and the product was purified using gel-filtration. The elution pattern showed that fractions 18–32 contained carbohydrates (part of tremella glycoprotein), as detected with phenol-sulfuric acid, but no salts (chemical reagents) were detected in those fractions and the salts detected by the analysis of electric conductivity were eluted from the column after fraction 34. Thus, iodinated TGP (ITG) was obtained and its IR spectra and iodine content were analyzed. IR spectra of TGP and ITG were recorded and compared. The main peaks were observed at 3363, 3267, 1631, 1527, 1396, 1303, 1087, 902, 810, 624, and 536 cm^−1^ in the spectrum of ITG and at 3309, 2931, 1462, 1381, 1319, 1261, 1084, 1022, 729, and 624 cm^−1^ in the TGP spectrum. ITG showed a strong peak at 536 cm^−1^, which is the typical peak of halogenated hydrocarbons, suggesting that the iodination was successful. The iodine content in TGP and ITG was detected using ICP-MS and compared. No iodine could be detected in TGP, whereas ITG contained 0.28% of iodine providing additional evidence of successful iodination and the sample was used in the methodology studies.

The stability of iodine labels is very important for getting accurate results when detecting iodinated sugar complexes in biological samples. The label stability was tested in cell culture. Small molecules, including free iodine, were isolated and collected from the cell culture using a Sephadex G-50 column, and the collected fractions were evaporated to determine iodine content. No free iodine was detected in the collected fractions using ICP-MS (data not shown), compared with cell culture, regardless of the incubation time (1, 2, or 3 h). Then the blood samples collected from the administrated ITG rats was dissolved in pure water and was applied to a Sephadex G-50 column to collect small molecule fractions. No free iodine was detected in the collected fractions using ICP-MS (data not shown). Those results suggesting that the iodine-labeled ITG is stable in biologic samples.

### 3.3. Studies in Methodology

In order to establish the method of detection for ITG in biological samples, we examined various aspects of our methodology, including treatment of samples, linearity and linear range, accuracy, stability, and sample recovery rate. 

#### 3.3.1. Treatment of Samples

Methods that could be employed in order to release iodine from biological samples prior to iodine detection by ICP-MS include microwave, acid, and alkali digestion. We tested these three methods and found the iodine values in differently digested samples to be similar in the ICP-MS assay (data not shown). However, as the alkali digestion is stable and convenient to perform, it was used for the rest of the study.

#### 3.3.2. Linearity Study

In the study of linearity and linear range of the method, 1 mg/mL I^−^, as standard iodide solution, was diluted to 1 ng/mL, 5 ng/mL, 10 ng/mL, 50 ng/mL, 100 ng/mL, and 300 ng/mL standard solutions with 1% TMAH. The content of iodine was detected by ICP-MS according to the conditions described in the Methods Section.

**Figure 3:**
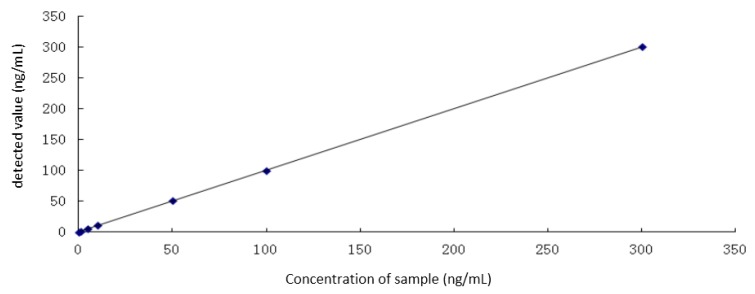
Standard curve for iodine.

The standard curve (Figure 3) (*y* = 1.0006*x* + 0.39141, R = 0.9998) showed satisfactory linearity in the range of 0–300 ng/mL.

#### 3.3.3. Evaluation of Accuracy

For evaluation of accuracy, ITG (50 mg) was digested and diluted to 2500 times, then 1 mL of the diluted solution was tested 10 times by ICP-MS. The results (Table 3) suggested adequate accuracy of the method.

#### 3.3.4. Evaluation of Repeatability

The repeatability of the method was examined as follows: seven 50 mg samples of ITG were prepared. The samples were digested and diluted 1000 times and 1 mL diluted solution was taken to detect its iodine content by ICP-MS. The content of iodine in each ITG sample was calculated from the detected results and concentration of the sample. The results are listed in Table 4. The RSD calculated from the detection data shows good repeatability of the method. 

#### 3.3.5. Evaluation of Method Stability

ITG (50 mg) was digested according to the procedure outlined in the Methods section and detected by ICP-MS every 2 h. The results suggest that the method is stable within 8 h (Table 5).

#### 3.3.6. Recovery Rate Evaluation

The sample recovery rate was tested. Six sample copies containing 50 mg ITG in 3.5 mL ultrapure water were mixed with 0.5 mL of 1 mg/mL NaI aqueous solution (equal to 0.42 mg I^−^). The sample digestion and detection with ICP-MS were performed as described previously in the Methods section. The results of recovery rate evaluation are shown in Table 6, and indicate that the method is feasible.

### 3.4. Detection of ITG in Biological Samples

#### 3.4.1. ITG Content in Rat Blood

Another batch ITG which containing 0.55% iodine was used for preparing the biological samples. Blood samples (0.5 mL) were digested, diluted (25 times), and 1 mL diluted solution was take out to detect the content of iodine with ICP-MS. The concentration of iodine was read from the instrument directly, and the content of glycoprotein calculated from the content of iodine in ITG (Table 7). The total amount of ITG in rat blood (12.6 mL/rat) was 5.92, 9.56, and 10.51 mg after 1, 2, and 3 h, indicating 2.96%, 4.78%, and 5.3% of ITG present, at each respective time point.

#### 3.4.2. ITG Content in Rat Organs

Powdered, freeze-dried organs (100 mg) were digested and their iodine content determined using ICP-MS and the content of ITG in organs were calculated from the content of iodine in ITG and the weight of whole organ (Table 8).

## 4. Discussion and Conclusion

The results show that ITG was slowly absorbed into the blood stream after oral administration, reaching the highest plasma concentration (approximately 5%) after 3 h. Five main organs contained 4.3% ITG 3 h after oral administration. Nearly half of the ITG was distributed in the liver, with significant amounts of ITG also found in the kidney and lung. In total, approximately 10% of ITG could be found in the rat body, suggesting that the method could detect and trace glycoproteins in the animal body.

Glycoproteins are easily labeled with iodine, forming stable derivatives in animal bodies. The iodinated glycoproteins can be detected in biological samples using ICP-MS, after digestion. The methodology studies revealed that the proposed method is sensitive (>ng level), stable, and easy to perform, and could be widely used in the study of glycoprotein absorption, distribution, and elimination. 

## Figures and Tables

**Table 1:** Properties of fractions A, B, C, and D.

Properties	A	B	C	D
Carbohydrates (%)	63.2	70.3	78.5	85.7
Proteins (%)	23.4	21.7	13.6	3.6
Molecular weight (KDa)	30.8	10.4	6.8	3.1

**Table 2:** ICP-MS instruments working conditions of reference.

Instrument Parameters	Numerical Values	Units
Radio frequency power	1550	W
Sampling cone/intercept cone	1/0.4	mm
Sampling dept	8	mm
Atomizing chamber temperature	2	°C
Collision response gas pool	5.0	mL/min
Internal standard to join	Online join the standard	
The plasma flow	15	L/min
The dilution air flow	0.60	L/min
Scanning method	Jump peak	
The carrier gas flow rate	0.74	L/min

**Table 3:** Results of the accuracy experiment on ITG *(n* = 10).

Detected Value (ng/mL)										$\bar{x}$	RSD (%)
>1	>2	>3	>4	>5	>6	>7	>8	>9	>10		
117.1	107.3	112.7	108.3	106.0	106.1	113.0	115.7	110.7	105.0	110.2	3.9

**Table 4:** Results of the repeatability experiment on ITG.

Content of Iodine in ITG (μg/50 mg)							$\bar{x}$	RSD (%)
>1	>2	>3	>4	>5	>6	>7		
282	277	286	283	301	279	271	283	4.5

**Table 5:** Results of the stability experiment on ITG.

Time of Detection (h)	Concentration of Iodine (ng/mL)
0.0	118.6
2.0	113.5
4.0	116.9
8.0	122.9
$\bar{x}$	125.5
RSD (%)	4.5%

**Table 6:** The results of sample recovery rate.

Samples	Weight of Sample (mg)	Iodine Content in 50 mg of Sample (μg)	Amount of Added Iodine (μg)	Detected Amount of Iodine (μg)	Recovery Rate (%)	RSD (%)
1	50	276	42	689	99.0	5.2
2	50	276	42	676	97.1	
3	50	276	42	705	101.2	
4	50	276	42	663	95.2	
5	50	276	42	669	96.1	
6	50	276	42	646	92.8	

**Table 7:** Content of iodine and ITG in rat blood at different times.

Time Points	Detected Concentration of Iodine (ng/mL)	Content of Iodine in Blood Samples (ng/mL)	Total Amount of ITG in Rat Blood (mg)	Absorbed Amount of ITG in Rat Blood (%)
1h	51.7 ± 3.5	2583.8	5.9	3.0
2h	83.5 ± 5.7	4175.9	9.6	4.8
3h	91.8 ± 6.4	4588.1	10.5	5.3
Blank	1.2 ± 0.4	－	－	－

**Table 8:** The content of iodine and ITG in rat organs.

Organs	Detected Amount of Iodine in Sample Group (ng/100 mg)	Detected Amount of Iodine in Blank Group (ng/100 mg)	Content of Iodine in Sample Group (ng/100 mg)	Content of ITG in Sample Group (ng/100 mg)	Content of Iodine in Organs (ng)
Heart	782 ± 25	658 ± 22	124	22,572	39,000
Liver	822 ± 32	230 ± 19	591	107,454	1,922,000
Spleen	1328 ± 42	380 ± 21	948	172,363	271,000
Lung	1971 ± 49	301 ± 16	1670	303,636	103,000
Kidney	1556 ± 35	431 ± 29	1124	204,363	107,000
